# A temperature dependent pilin promoter for production of thermostable enzymes in *Thermus thermophilus*

**DOI:** 10.1186/s12934-023-02192-1

**Published:** 2023-09-19

**Authors:** Lennart Kirchner, Volker Müller, Beate Averhoff

**Affiliations:** Molecular Microbiology & Bioenergetics, Institute of Molecular Biosciences, Johann Wolfgang Goethe University Frankfurt/Main, Max-von-Laue- Str. 9, 60438 Frankfurt, Germany

**Keywords:** Gene expression, Thermophiles, Promoters, *Thermus thermophilus* HB27, Thermozymes

## Abstract

**Background:**

Enzymes from thermophiles are of great interest for research and bioengineering due to their stability and efficiency. Thermophilic expression hosts such as *Thermus thermophilus* [*T. thermophilus*] can overcome specific challenges experienced with protein production in mesophilic expression hosts, such as leading to better folding, increased protein stability, solubility, and enzymatic activity. However, available inducible promoters for efficient protein production in *T. thermophilus* HB27 are limited.

**Results:**

In this study, we characterized the *pilA4* promoter region and evaluated its potential as a tool for production of thermostable enzymes in *T. thermophilus* HB27. Reporter gene analysis using a promoterless β-glucosidase gene revealed that the *pilA4* promoter is highly active under optimal growth conditions at 68 °C and downregulated during growth at 80 °C. Furthermore, growth in minimal medium led to significantly increased promoter activity in comparison to growth in complex medium. Finally, we proved the suitability of the *pilA4* promoter for heterologous production of thermostable enzymes in *T. thermophilus* by producing a fully active soluble mannitol-1-phosphate dehydrogenase from *Thermoanaerobacter kivui* [*T. kivui*], which is used in degradation of brown algae that are rich in mannitol.

**Conclusions:**

Our results show that the *pilA4* promoter is an efficient tool for gene expression in *T. thermophilus* with a high potential for use in biotechnology and synthetic biology applications.

**Supplementary Information:**

The online version contains supplementary material available at 10.1186/s12934-023-02192-1.

## Background

Enzymes from thermophiles have attracted significant interest for research and bioengineering application due to their high stability and high catalytic efficiency [1–3]. The production of thermostable enzymes used in these fields is most commonly performed in mesophilic expression hosts, for example *Escherichia coli* [*E. coli*], *Bacillus subtilis* [*B. subtilis*] or *Pichia pastoris* [*P. pastoris*] [4–6]. Although the heterologous production of thermostable enzymes in mesophilic expression hosts can be successful, often the folding of the proteins of thermophilic bacteria is much better at high production rate in thermophilic expression hosts. It was recently shown that thermostable enzymes can be folded very differently when produced in mesophilic expression hosts, which can influence activity and thermostability [7]. Some thermostable enzymes are not active at all, when produced in mesophilic expression hosts [8]. Moreover, problems arising from the absence of cofactors or chaperones in mesophilic hosts have to be overcome [9–11]. The latter might affect the incorporation of cofactors such heme or iron sulfur clusters. Furthermore, thermostable enzymes can form aggregates known as inclusion bodies when produced in mesophilic expression hosts [8].

The use of thermophilic expression hosts and the development of genetic tools for the overproduction of thermostable enzymes is a popular strategy to circumvent these problems [1, 12, 13]. Recent studies highlighted the potential of the fungus *Chaetomium thermophilum* [*C. thermophilum*] and the hyperthermophilic archaeon *Sulfolobus solfatarius* [*S. solfatarius*] as possible thermophilic expression hosts [14, 15]. In this study we focus on *Thermus thermophilus* [*T. thermophilus*] as thermophilic expression host. *T. thermophilus* is a thermophilic bacterium that grows optimally at high temperatures between 55 and 80 °C with high growth rates leading to high cell mass [16]. Another outstanding trait which makes *T. thermophilus* a highly suitable candidate as production platform for thermostable enzymes is its high frequency of natural transformation supporting the use of this organism as cell factory, but also as an expression host for directed evolution studies or for the construction of genetic libraries and genomic and metagenomic studies [17–19]. However, although *T. thermophilus* HB27 is a popular model organism, the pool of strong, regulable promoters for efficient protein production is still limited. Three inducible promoters, namely the *P*_*arg*_, *P*_*dnaK*_ and *P*_*scs−mdh*_ promoters were characterized and two systems for efficient overproduction were reported [20–22]. One utilizes the *P*_*nar*_-promoter from *T. thermophilus* HB8, a close relative to *T. thermophilus* HB27 which is induced by anaerobic conditions and the presence of nitrate; *T. thermophilus* HB27 does not perform nitrate respiration due to a lack of the genes involved [21, 23]. Another system uses a silica inducible promoter from *T. thermophilus* HB8 which is induced by addition of 10 mM silica. However, the latter led to a significant growth inhibition [22]. The problems encountered during expression of fully active enzymes from thermophiles in mesophilic expression hosts together with very limited number of thermophilic expression hosts prompted us to develop a novel expression system for *T. thermophilus* as thermophile expression host.

For the adhesion on solid surfaces and for the uptake of DNA from the environment *T. thermophilus* requires type IV pili (T4P) which consist of the major structural subunit PilA4 [24, 25]. For the formation of long pilus structures a high number of PilA4 subunits is required. This led to the suggestion that the *pilA4* gene must be highly expressed under T4P-forming conditions. In previous studies we found that the growth temperature had a significant effect on T4P production and on *pilA4* expression such as higher amounts of *pilA4* transcript were detected during growth at 68 °C compared to 80 °C [26]. This indicates that the *pilA4* promoter undergoes thermoregulation which can be mediated by many different factors in bacteria such as riboswitches, RNA-thermometers, temperature dependent transcription factors or heat shock proteins [27–29].

In this study we describe the use of the promoter of the *pilA4* gene for temperature dependent production of thermostable β-glucosidase in *T. thermophilus* and fully active and soluble mannitol-1-phosphate dehydrogenase from the anaerobic thermophilic bacterium *Thermoanaerobacter kivui* [*T. kivui*]. Taken together this novel expression system can be used for the production of different enzymes from thermophilic and mesophilic bacteria and is a very promising system for advancing biotechnological applications.

## Results and discussion

### Construction of a P_pilA4_ expression vector

**Fig. 1:**
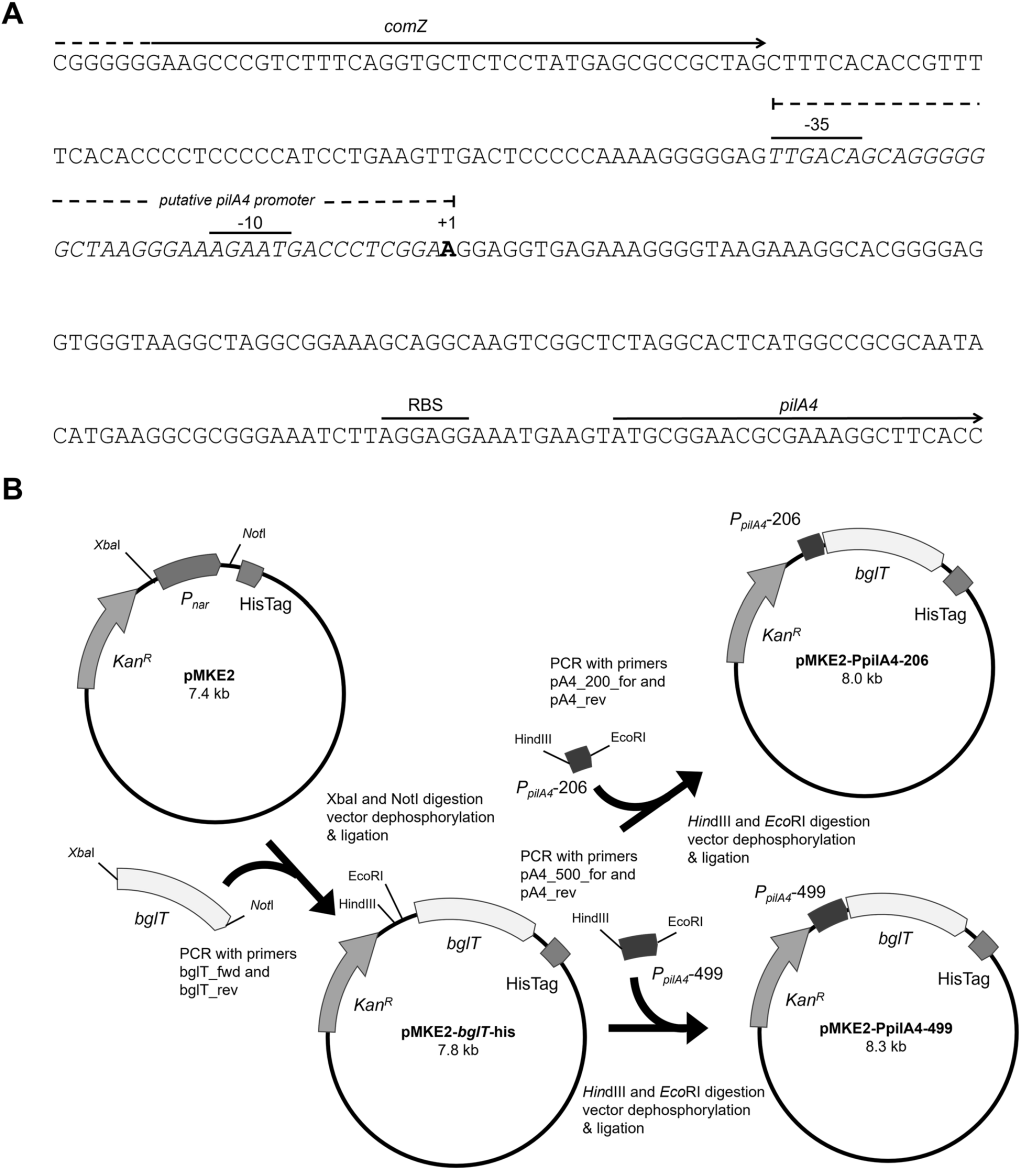
Sequence of the intergenic region between *comZ* and *pilA4* and plasmid construction. **A** The − 35 and − 10 regions of the predicted *P*_*pilA4*_-promoter, start of the transcript (+ 1) and the potential ribosome binding site (RBS) are indicated above the sequences. Arrows denote the transcriptional orientation of *comZ* and *pilA4*. **B** The plasmid pMKE2, which encodes for a kanamycin resistance gene (Kan^R^), was digested using *Xba*I and *Not*I to eliminate the *P*_*nar*_-fragment. The promoterless *bglT* gene amplified by PCR using genomic DNA of *T. thermophilus* HB27 was introduced into the *Xba*I and *Not*I restriction sites. The stop codon was omitted to generate a transcriptional fusion with the hexahistidin tag encoded by pMKE2 (HisTag). The two *P*_*pilA4*_ DNA fragments amplified from *T. thermophilus* genomic DNA by PCR were introduced *via Eco*RI and *Hin*dIII restriction sites

We previously reported that *T. thermophilus* produces two different forms of pili differing in structure and protein composition, wide T4P structures comprising of the pilin PilA4, and narrow pilus structures formed by PilA5. The latter is essential for twitching motility [24]. PilA4 was found to be essential for natural transformation and for assembly of both the wide and narrow pili [24]. To identify the potential promoter region of *pilA4* we performed an *in silico* analysis of the DNA region upstream of *pilA4* using the software phiSITE PromotorHunter [30]. A potential – 35 and – 10 region was detected 131–170 bp upstream of the *pilA4* start codon (Fig. 1A). To examine the promoter activity of this region a 206 bp DNA-fragment spanning – 25 to – 231, relative to the translation start site of *pilA4* was cloned into pMKE2-*bglT*-his (Fig. 1B). Agarose gel electrophoresis analysis of the plasmid construction and verification by restriction analysis is included in Additional file 1: Fig. S1. The detected DNA-fragments corresponded to the expected sizes and sequence analysis of the inserted fragments showed that they were inserted correctly. The temperature dependent *pilA4* transcript levels detected in *T. thermophilus* in previous analyses suggested that the *pilA4* expression undergoes temperature-dependent transcriptional regulation [26]. In order to include potential distantly located binding sites of proteins involved in temperature-dependent regulation of the *pilA4* promoter a longer 499 bp DNA region spanning − 25 to -524 bp upstream of the *pilA4* start codon was also cloned into pMKE2-*bglT*-his. The plasmid construction scheme is shown in Fig. 1B. The two plasmids, designated pMKE2-PpilA4-206 and pMKE2-PpilA4-499 were transformed into *T. thermophilus* Δ*bglT via* natural transformation.

###  Effect of fragment length on β-glucosidase expression

**Fig. 2:**
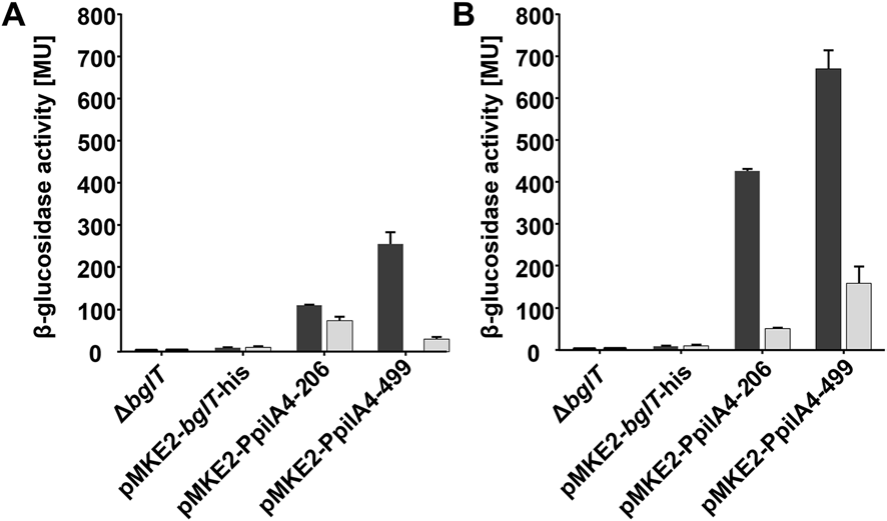
*P* *pilA4* promoter activities. β-glucosidase activities in *T. thermophilus* Δ*bglT*, *T. thermophilus* Δ*bglT* (pMKE2-*bglT*-his), *T. thermophilus* Δ*bglT* (pMKE2-PpilA4-206) and *T. thermophilus* Δ*bglT* (pMKE2-PpilA4-499) grown in TM^+^-medium at 68 °C (black bars) and 80 °C (grey bars) respectively **A**. β-glucosidase activities in *T. thermophilus* Δ*bglT*, *T. thermophilus* Δ*bglT* (pMKE2-*bglT*-his), *T. thermophilus* Δb*glT* (pMKE2-PpilA4-206) and *T. thermophilus* Δ*bglT* (pMKE2-PpilA4-499) grown in minimal medium with 20 mM pyruvate as carbon sorce at 68 °C (black bars) and 80 °C (grey bars) **B**. The β-glucosidase activities are given in Miller units (MU). Values are expressed as means ± standard deviations (n = 3)

To analyze the promoter activities reporter gene analyses were performed. Therefore, *T. thermophilus* (pMKE2-PpilA4-206) and *T. thermophilus* (pMKE2-PpilA4-499) were grown in TM^+^-medium at optimal growth temperature (68 °C) to mid exponential phase and the β-glucosidase activities were determined. Both recombinant strains exhibited β-glucosidase activities significantly higher than *T. thermophilus* Δ*bglT* and the control strain carrying a promoterless pMKE2-*bglT-*his plasmid (Fig. 2A, black bars). This leads to the conclusion that both, the 206 bp and the 499 bp region convey promoter activity. Interestingly, in *T. thermophilus* Δ*bglT* (pMKE2-PpilA4-499), β-glucosidase activities of ~ 270 MU were detected, whereas in *T. thermophilus* Δ*bglT* (pMKE2-PpilA4-206) a significantly lower level of activity of ~ 110 MU was detected (Fig. 2A). This suggests that the 499 bp DNA-fragment spanning the – 10 and – 35 region of the *P*_*pilA4*_ promoter contains regulatory regions important for optimal *P*_*pilA4*_ promoter activity.

### Effect of growth temperature on β-gucosidase expression

In former studies we found that elevated growth temperatures of 80 °C significantly decreased *pilA4* transcript levels [26]. To analyze the *P*_*pilA4*_ promoter activities at 80 °C *T. thermophilus* Δ*bglT* (pMKE2-PpilA4-206) and *T. thermophilus* Δ*bglT* (pMKE2-PpilA4-499) were grown to mid-exponential growth phase in TM^+^-medium at 80 °C. The β-glucosidase activities in *T. thermophilus* Δ*bglT* (pMKE2-PpilA4-499) were ~ 30 MU which is significantly lower than those detected in cells grown at 68 °C (~ 270 MU) and in the range of the β-glucosidase activities in the control strain *T. thermophilus* Δ*bglT* (pMKE2-*bglT-*his) (~ 10 MU) (Fig. 2A). To ensure that the low β-glucosidase activities in *T. thermophilus* Δ*bglT* (pMKE2-PpilA4-499) were not due to plasmid loss at this high temperature, we analyzed the plasmid integrity by retransformation into *E. coli* TOP10 cells and found that the plasmid was successfully retransferred into *E. coli*. Analyses of the β-glucosidase activities in cells of *T. thermophilus* Δ*bglT* (pMKE2-PpilA4-206) grown at 80 °C also led to the detection of a decrease in activities (~ 73 MU) in comparison to cells grown at 68 °C (~ 110 MU) (Fig. 2A). However, the decrease in β-glucosidase activities was not as dramatic as in *T. thermophilus* Δ*bglT* (pMKE2-PpilA4-499). This suggests that the shorter ~ 200 bp *P*_*pilA4*_ region is also subject to temperature-dependent regulation but not as effectively as the longer fragment. This might be due to missing binding sites for bacterial enhancer binding proteins (bEBPs) which can be located quite distantly from the promoter sequence. Such bEBPs effect transcription of the pilin gene through topological changes in the DNA and interaction with the RNA-polymerase holoenzyme. Although so far only very little is known about regulation of T4P-related genes in *T. thermophilus*, bEBPs have been shown to be involved in the regulation of these genes in other T4P forming organisms such as *Pseudomonas aeruginosa* [*P. aeruginosa*] or *Geobacter sulfurreducens* [*G. sulfurreducens*] [26, 31, 32]. Complex regulatory mechanisms even including multiple promotors involving bEBPs have been previously identified for the regulation of major pilin subunit genes in *Neisseria gonorrhoeae* [*N. gonorrhoeae*] [33].

### Effect of growth medium on β-glucosidase expression

In former studies we reported that growth in minimal medium led to significantly increased *pilA4* transcript levels [26]. We next examined whether our expression system was affected by growth in minimal medium by analyzing the β-glucosidase activities in cells of *T. thermophilus* Δ*bglT* (pMKE2-PpilA4-499) throughout growth at 68 and 80 °C, respectively in minimal medium with pyruvate as carbon source (Fig. 2B). Growth in minimal medium at 68 °C led to significantly increased β-glucosidase activities of maximally ~ 700 MU (Fig. 2B, black bars) in comparison to ~ 270 MU detected in cells grown in TM^+^ medium. Growth in minimal medium at 80 °C also led to increased β-glucosidase activities of maximally ~ 350 MU in comparison to the activities of ~ 30 MU after growth at 80 °C in TM^+^ medium (Fig. 2B, grey bars). Taken together, these findings lead to the conclusion that both, growth temperature and medium have a significant effect on gene expression mediated by the *P*_*pilA4*_ promoter with maximal gene expression during growth at 68 °C in minimal medium.

###  Effect of growth phase on β-glucosidase expression

**Fig. 3:**
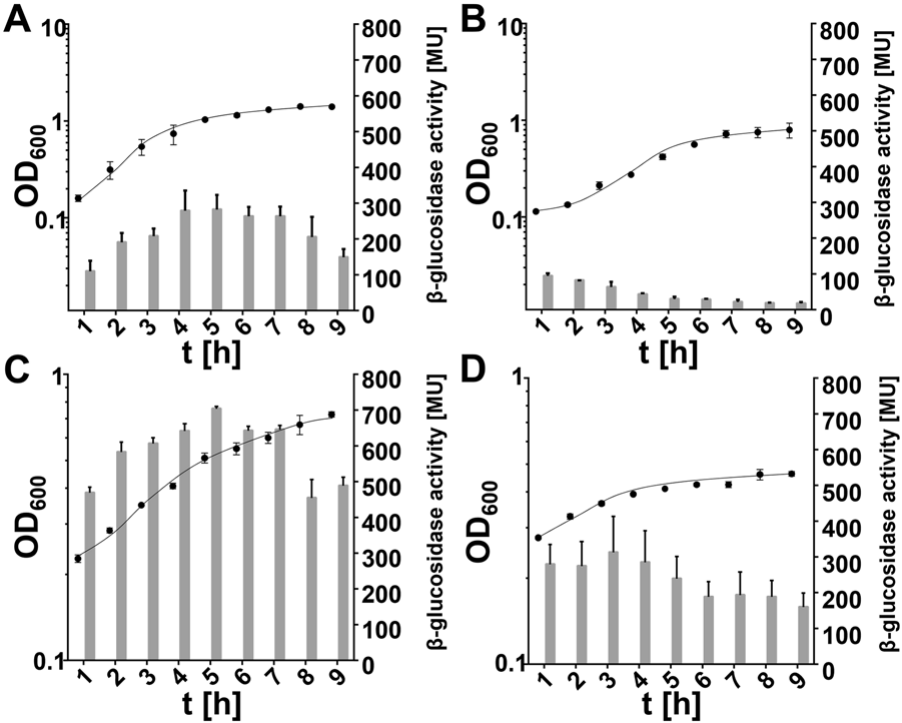
Growth-phase dependent β-glucosidase activities in cells. Shown are the optical density (•) and β-glucosidase activities (bars) of *T. thermophilus* Δ*bglT* (pMKE2-PpilA4-499) cells grown in TM^+^-medium at 68 °C (**A**), 80 °C (**B**) or in minimal medium with 20 mM pyruvate as carbon and energy source at 68 °C (**C**) or 80 °C (**D**)

In former studies it was reported that *T. thermophilus* HB27 is transformable in all growth phases, with lowest transformation frequencies in stationary growth phase [34]. To address the effect of growth phase on BglT production we examined the activities throughout growth in TM^+^ medium and minimal medium at 68 or 80 °C by analyzing the β-glucosidase activities in *T. thermophilus* Δ*bglT* (pMKE2-PpilA4-499) (Fig. 3). As shown in Fig. 3A, growth at 68 °C in TM^+^ medium led to exponential growth (µ = 0.93) directly after inoculation of the culture. A final OD_600_ of 1.4 was reached in the stationary phase after 9 h. A maximal β-glucosidase expression of 300 MU was detected in late exponential growth phase. Growth at 80 °C in TM^+^ medium led to a prolonged lag phase of 3 h followed by an exponential phase with a doubling time of 0.30 h^−1^ (Fig. 3B). A final OD_600_ of 0.9 was reached in stationary phase 9 h after inoculation. During growth at 80 °C in TM^+^ medium very low β-glucosidase activities of 100 MU were detected in the lag phase which even decreased throughout growth down to 30 MU in stationary growth phase.

Inoculation of fresh minimal medium at 68 °C with *T. thermophilus* Δ*bglT* (pMKE2-PpilA4-499) led to exponential growth directly after inoculation (Fig. 3C), with a growth rate of µ = 0.19 and a final optical density of 0.9 after 9 h. Maximal β-glucosidase activities of 700 MU were detected during exponential growth. Growth in minimal medium at 80 °C led to a reduced doubling time of 0.1 h^−1^ and a lower final optical density of 0.38. The measured β-glucosidase activities decreased throughout growth at 80 °C in minimal medium to a minimum of 150 MU in stationary phase (Fig. 3D). Taken together, highest β-glucosidase activities of 700 MU were detected during growth in minimal medium at 68 °C quite rapidly after only 5 h of cultivation, which underlines the suitability of this expression system for biotechnological applications, where time can be an important factor.

### Homologous production of BglT-his in T. thermophilus

**Fig. 4:**
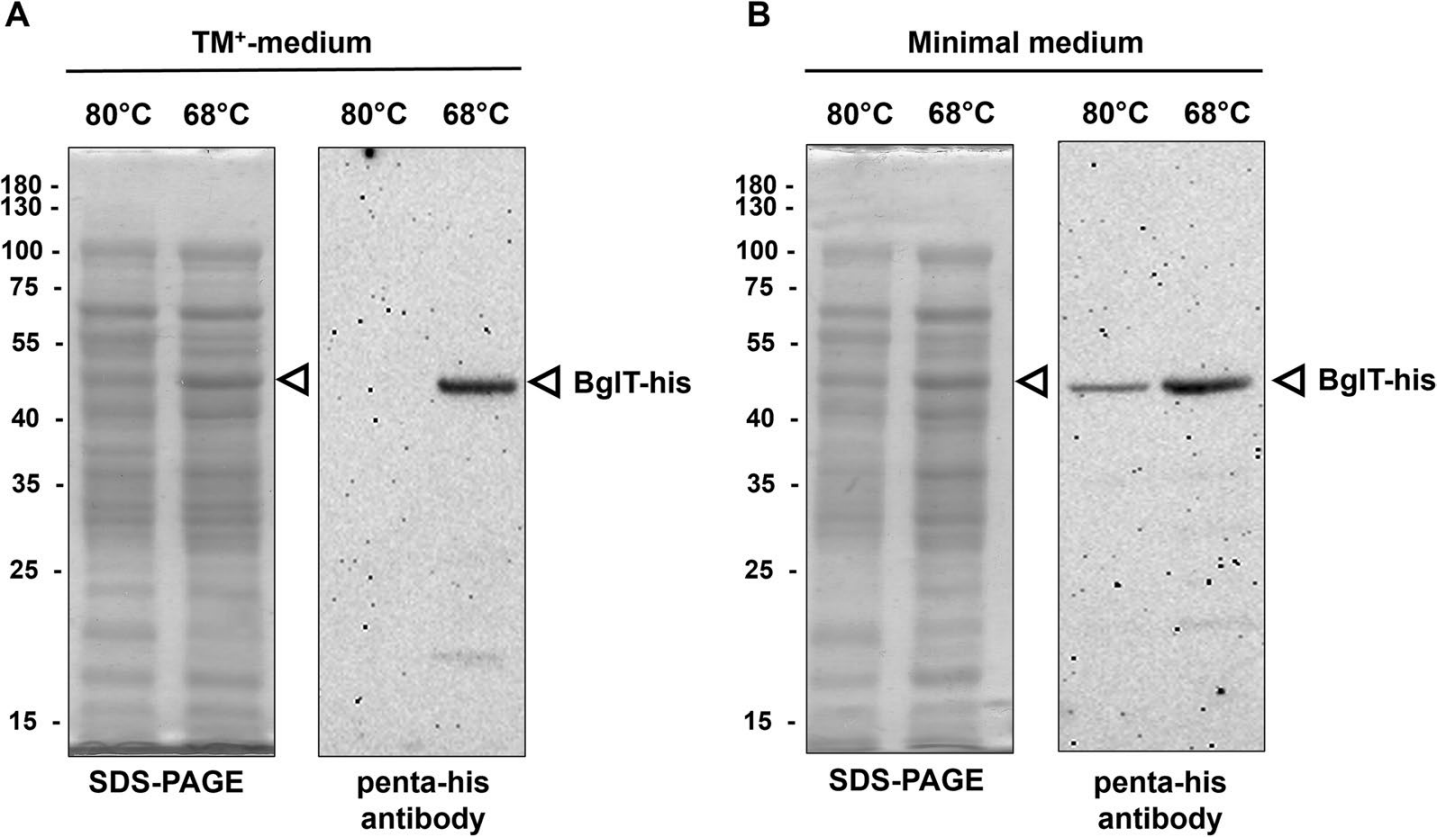
SDS-PAGE and Western blot analysis of homologous production of BglT-his in *T. thermophilus*. *T. thermophilus* Δb*glT* (pMKE2-*bglT*-his) cells were grown in 50 mL TM^+^ or minimal medium at either 68 or 80 °C, harvested in the mid exponential growth phase followed by cell lysis and seperation of cell extracts *via* SDS-PAGE. Each lane contains 10 µL of cell extracts normalized to OD_600_ = 10. Proteins were detected by Coomassie brilliant blue staining (**A**). Western blot analyses were performed using the penta-his antibody (1:10000 dilution) (**B**). The white arrow indicates the BglT-his

Next, we analyzed whether our *P*_*pilA4*_-promoter based gene expression system can indeed be used to induce protein production in *T. thermophilus*. Therefore, *T. thermophilus* Δ*bglT* (pMKE2-PpilA4-499) was cultivated in TM^+^ medium or minimal medium at 68 or 80 °C and harvested in the exponential growth phase. Western blot analyses were performed using the penta-his-antibody. After growth in TM^+^ or minimal medium at 68 °C both conditions his-tagged BglT was detected in amounts visible in the Coomassie stained SDS-PAGE and also in the Western blot (Fig. 4). As expected no BglT was detected after growth in TM^+^ medium at 80 °C (Fig. 4A) whereas small amounts of BglT were detected after growth in minimal medium at 80 °C (Fig. 4B).

### Heterologous production of MtlD-his from T. kivui

**Fig. 5:**
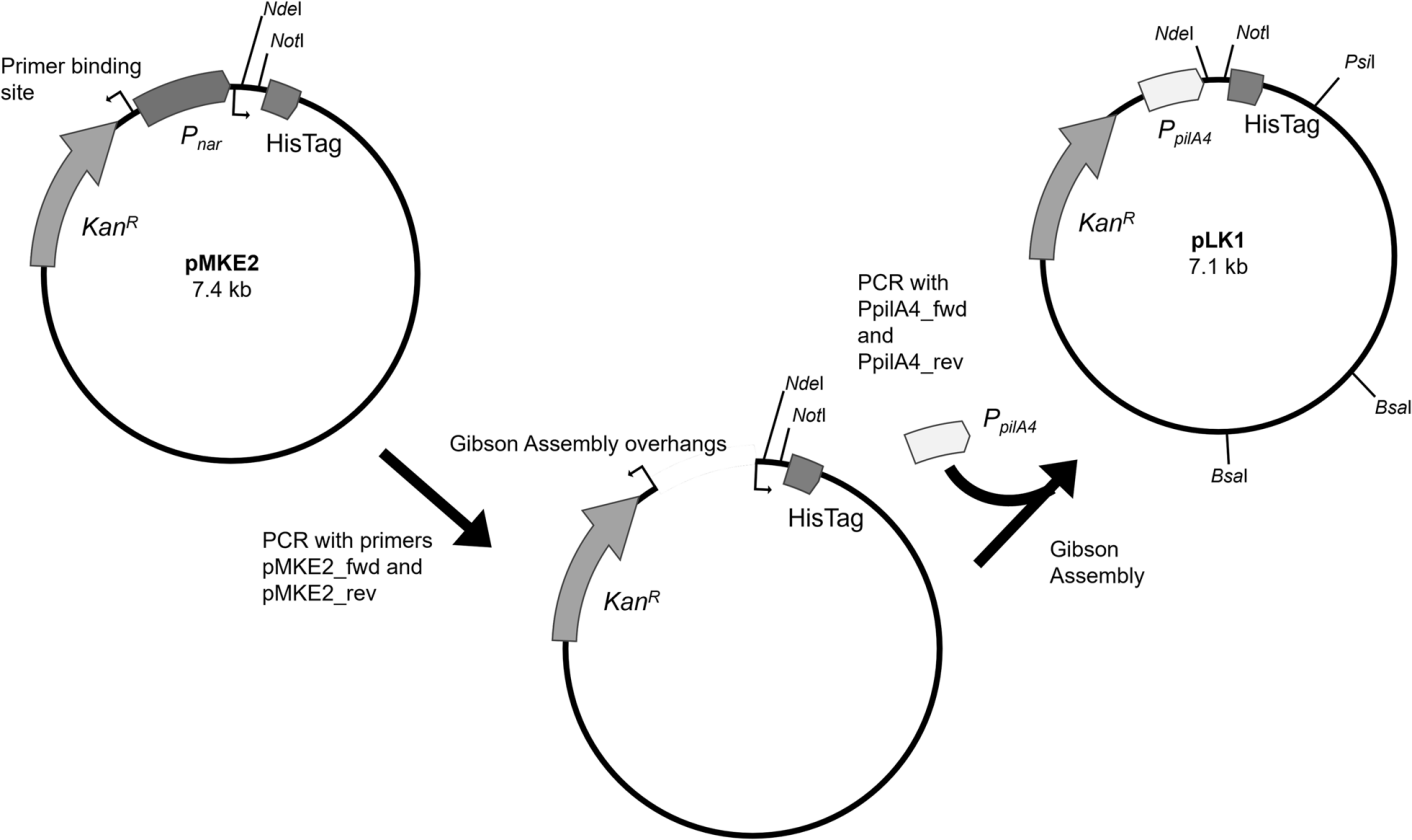
Generation of pLK1. The *P*_*nar*_-fragment was deleted from pMKE2 *via* PCR and the *P*_*pilA4*_ DNA fragment amplified by PCR was inserted by Gibson assembly leading to the expression plasmid pLK1.

To evaluate the suitability of our system for heterologous protein production, we used the mannitol-1-phosphate dehydrogenase MtlD from *T. kivui*. Therefore, we first generated pLK1, an expression vector utilizing the *pilA4* promoter region as displayed in Fig. 5. The *P*_*pilA4*_ containing DNA fragment with a length of 0.56 kb and the vector amplified by PCR with a length of 6.64 kb were detected by agarose gel electrophoresis (Additional file 1: Fig. S2A, B). pLK1 was verified by restriction analysis using *Bsa*I and *Psi*I. The length of the detected DNA-fragments (4.19 kb, 2.08 kb and 8.88 kb) corresponds to the expected length (Additional file 1: Fig. S2C).

The wildtype *mtlD* gene was cloned into pLK1 and transformed into *T. thermophilus* cells, but protein production was not observed (data not shown). This phenomenon has been observed with other proteins as well, likely due to the distinct codon usage of *T. thermophilus*, which has a high GC content of 68% compared to *T. kivui* with a GC content of 35%. To circumvent this problem we used a codon optimized *mtlD* gene. We cloned the codon-optimized gene into pLK1 to generate pLK1-*mtlD*-his which was transformed into *T. thermophilus* wildtype cells. pLK1-*mtlD*-his was verified *via* restriction analysis using *Nde*I and *Not*I. The lenght of the detected DNA-fragments (7.1 kb and 1.17 kb) corresponds to the expected length (Additional file 1: Fig. S2D).

**Fig. 6:**
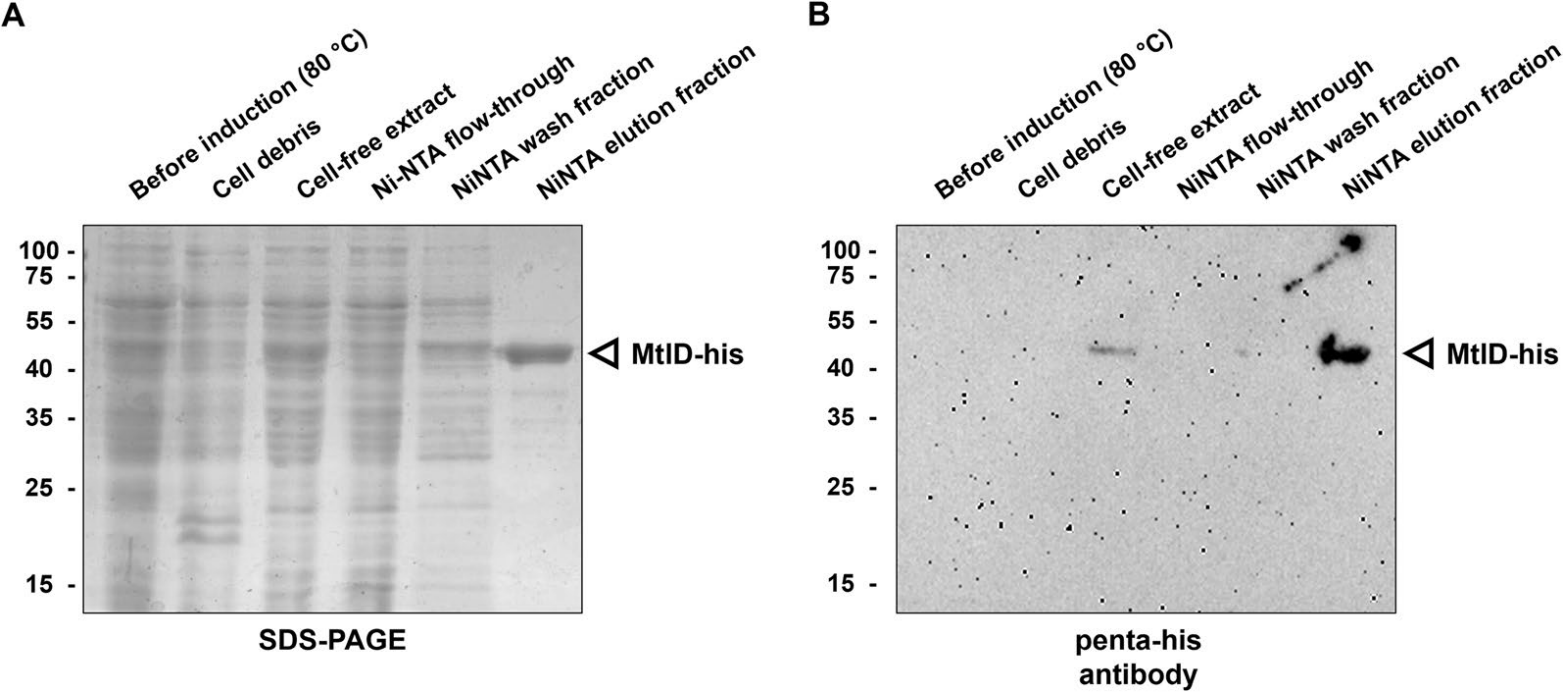
Purification of MtlD from *T. kivui* produced in *T. thermophilus*. The enzyme was purified by Ni-NTA (elution with 300 mM imidazole) and analyzed on a 12% SDS gel. The protein was stained with Coomassie brilliant blue R-250 (**A**). Western blot analysis of the fractions obtained during purification of MtlD-his. Western blot analysis was performed using the penta-his antibody (1:1000 dilution) (**B**). The white arrow indicates MtlD-his.

Cells were grown until mid exponential growth phase (OD_600_ = 0.6) in minimal medium, harvested and MtlD-his was purified *via* Ni-NTA affinity chromatography (Fig. 6). A protein yield of 9 mg MtlD per liter of culture was obtained, which is comparable to the protein yields achieved for heterologous protein production in *T. thermophilus* using the *P*_*sip*_- system which depends on a silica inducible promoter [22]. In contrast to the production of MtlD in *E. coli*, which was previously reported by Moon et al. [35], no MtlD was detected in the cell debris (Fig. 6B). Which indicates that the enzyme is fully soluble in the expression host *T. thermophilus*.

To assess the functionality of the purified enzyme, we analyzed MtlD-dependent fructose-1-phosphate reduction. The enzyme displayed specific activities of ~ 800 µmol min^−1^ mg^−1^ which were comparable to the enzyme purified from *E. coli* BL21 (DE3). These results demonstrate our *pilA4* promoter-based expression system can be used for heterologous production of thermostable, soluble and active proteins from other thermophilic bacteria in *T. thermophilus.* MtlD not only catalyzes the degradation of mannitol, as in *T. kivui*, but also the biosynthesis of mannitol-1-phosphate from fructose-6-phosphate. Mannitol has a wide range of applications in the food and pharmaceutical industry [36]. Our studies are the first step to establish a thermophilic production platform for mannitol.

## Conclusions

We reported the development of a temperature inducible *P*_*pilA4*_-based-expression system for homologous and heterologous production of thermostable proteins in *T. thermophilus*. We demonstrate the successful expression of soluble, fully active *T. thermophilus* β-glucosidase and *T. kivui* mannitol-1-phosphate dehydrogenase in *T. thermophilus.* We showed that the *pilA4*-promoter-dependent expression system is highly efficient yielding protein levels of 9 mg/L of culture. The high induction of the *pilA4* promoter at optimal growth temperature in exponential growth phase makes this novel expression system very well suitable for the fast production of high amounts of thermostable proteins for biochemical protein analysis and also for industrial applications. The promoter is regulated by an easy to perform temperature switch.

## Materials and methods

### Organisms and cultivation

*E. coli* TOP 10 was cultivated in LB “Luria-Bertani” complex medium (10 g/L tryptone, 10 g/L NaCl, 5 g/L yeast extract) at 37 °C and, when appropriate, 20 µg/mL kanamycin were added. *T. thermophilus* strains were grown at either 68 or 80 °C in TM^+^ medium (8 g/L tryptone, 4 g/L yeast extract, 3 g/L NaCl, 0.6 mM MgCl_2_ and 0.17 mM CaCl_2_) or *Thermus* minimal medium [37] with 20 mM pyruvate as carbon source. 20 or 40 µg/mL kanamycin in liquid or solid medium were added when appropriate.

### Plasmid construction

The primers and used in this study are presented in Additional file 1: Tables S1 and S2. Plasmid construction was carried out as displayed in Figs. 1B and 5. To generate a *pilA4*-promoter-dependent expression vector the plasmid pMKE2 [21] was used as backbone. First, the *P*_*nar*_ promoter was deleted from pMKE2 by *Xba*I and *Not*I digestion and replaced by the promoterless β-glucosidase gene *bglT* (TT_P0042, GenBank accession number AF135400.2), which was amplified from chromosomal DNA of *T. thermophilus* HB27 (GenBank accession number AE017221) with Phusion DNA polymerase (New England Biolabs, Ipswitch MA, USA) using the primers bglT_for and reverse primer bglT_rev (Fig. 1B). The DNA was denatured at 98 °C for 3 min, followed by 35 cycles of denaturation at 98 °C for 30 s, annealing at 60–80 °C for 30 s, and extension at 72 °C for the appropriate time depending on the desired product length assuming an optimal polymerase speed of 0.5 kb per minute. A final extension step was performed at 72 °C for 5 min. The resulting DNA fragment encodes a promoterless *bglT* gene devoid of the stop codon flanked by *Xba*I and *Not*I restriction sites. A 499 bp and 206 bp DNA fragment spanning the putative promoter region upstream of the pilin gene *pilA4* (TT_C0858, GenBank accession number AAS81202.1) from *T. thermophilus* was amplified *via* PCR using the two forward primers pA4_500_for and pA4_200_for, and the reverse primer pA4_rev, respectively. The PCR products were purified *via* the PCR CleanUP Kit (Merck, Darmstadt, Germany) digested with *Hin*dIII and *Eco*RI, and inserted into the pMKE2-*bglT*-his plasmid leading to the two recombinant plasmids pMKE2-PpilA4-206 and pMKE2-PpilA4-499, respectively (Fig. 1B). The plasmids were verified by restriction (Additional file 1: Fig. S1) and sequencing. The *P*_*pilA4*_-dependent expression was performed in a markerless Δ*bglT* mutant kindly provided by Prof. Dr. José Berenguer.

To produce MtlD from *T. kivui* in *T. thermophilus* HB27 using the *P*_*pilA4*_ promoter, the expression vector pLK1 was constructed by introducing a 499 bp DNA fragment of the *pilA4* promoter region into pMKE2, thereby replacing the *P*_*nar*_ fragment in front of the multiple cloning site (Fig. 5). For heterologous production of MtlD of *T. kivui* (TKV_c02860, GenBank accession number CP009170.1) in *T. thermophilus* a codon optimized *mtlD* gene was used. The codon optimization was performed by Genscript (Rijswijk, Netherlands) using their GeneSmart™ algorithm. The codon-optimized sequence is provided in Additional file 1: Table. S3. From Genscript we received the codon-optimized *mtlD* on the plasmid pUC57 (GenBank accession number Y14837.1). The *mtlD* gene was flanked by *Nde*I and *Not*I restriction sites which were used to insert it into pLK1, leading to the expression plasmid pLK1-*mtlD*-his. We verified pLK1 and pLK-*mtlD*-his by restriction analyses (Additional file 1: Fig. S2).

### β-Glucosidase reporter-gene assay

To analyze *bglT* production under the control of the *T. thermophilus pilA4* promoter the plasmids pMKE2-PpilA4-206 and pMKE2-PpilA4-499 were transferred into *T. thermophilus* Δ*bglT* by natural transformation. Therefore, exponentially grown cells were incubated with 500 ng of plasmid DNA for 3 h at 68 °C and then plated on TM^+^-agar containing 40 µg/mL kanamycin. The β-glucosidase activities were analyzed as previously described with slight modifications [38]. Cells containing either pMKE2-PpilA4-206 or pMKE2-P-pilA4-499 were grown in either TM^+^ medium or minimal medium at either 68 or 80 °C and harvested in different growth phases by centrifugation. After removal of the supernatant the cells were resuspended in 1 mL Z-buffer (8.8 mM Na_2_HPO_4_, 4.5 mM NaH_2_PO_4_, 100 mM KCl, 10 mM MgSO_4_ × 7H_2_O, 3 mM β-mercaptoethanol, pH 7) and treated with 10 µL toluol for 1 min at 80 °C. 200 µL of the permeabilized cells were transferred into 800 µL preheated (80 °C) Z-buffer. The β-glucosidase-reaction was started by addition of 50 µl 2-nitrophenyl-ß-D-glucopyranosid (2NPGlc) from initial 10 mg/mL stock solution (Carl Roth GmbH, Karlsruhe, Germany) and terminated by addition of 200 µL of 1 M Na_2_CO_3_ after a sufficient yellow color had developed. After centrifugation for 5 min at 16,000×g, absorption of the supernatant was recorded at 420 and 550 nm respectively. Miller units [MU] = 1000 × OD_420_ / reaction time (min) × reaction volume (ml) × OD_550_ were calculated according to Miller [39].

### Heterologous production and purification of MtlD-his

To produce MtlD of *T. kivui* in *T. thermophilus* HB27, 1 L of minimal medium was inoculated with *T. thermophilus* (pLK1-*mtlD*-his) to OD_600_ = 0.1. The cells were grown to mid exponential phase (OD_600_ = 0.6) at 68 °C and harvested by centrifugation. The cells were resuspended in 25 mL buffer 1 (50 mM Tris-HCl, 300 mM NaCl, pH = 7), disrupted *via* French press and cell debris was removed by centrifugation (14,000×g for 15 min). MtlD-his was purified *via* immobilized nickel ion chromatography using 3 mL of Ni-NTA resin in a 14 ml protino column (Macherey-Nagel, Düren, Germany). The cell-free extract was incubated with Ni-NTA for 1 h at 4 °C followed by washing with 60 ml buffer 1 containing 50 mM imidazole. MtlD-his protein was eluted with 6 mL buffer 1 containing 300 mM imidazole and concentrated in a Vivaspin® protein concentrator spin column (Sartorius, Göttingen, Germany; 10,000 Da cutoff). The protein concentration was determined according to Bradford [40]. Bovine serum albumin (BSA) was used as the standard protein. Standard solutions with known concentrations (0–90 µg/mL) were prepared. Samples were diluted 1:100 with buffer 1. 100 µL of the sample were mixed with 1 mL Bradford reagent (0.01% (w/v) Coomassie Brilliant Blue G-250, 4.7% (w/v) ethanol, 8.5% (w/v) H_3_PO_4_) in 1 mm plastic cuvettes (Sarstedt, Nümbrecht, Germany). The mixture was gently vortexed and incubated at room temperature for 10 min. Absorption was measured at 595 nm after calibrating the spectral photometer with plain Bradford solution. A calibration curve was generated by plotting the known BSA concentrations against their corresponding absorbance values. The equation of the standard curve was used to calculate the protein concentration of each unknown sample. All measurements were taken in triplicate.

### Western-blot analysis

*T. thermophilus* Δ*bglT* (pMKE2-PpilA4-499) and *T. thermophilus* (pLK1-*mtlD*-his) were grown in 50 mL TM^+^ or minimal medium at 68 or 80 °C. Cells were harvested in mid exponential growth phase (OD_600_ = 0.8) by centrifugation for 2 min at 16,000×g followed by lysis in SDS-sample buffer (4% (w/v) SDS, 12% (v/v) glycerol, 50 mM Tris-HCl, pH 6.8, 2% (v/v) mercaptoethanol, 0.01% (w/v) Serva Blue G, OD_600_ = 10) by boiling for 20 min. The samples (10 µg protein) were separated by SDS-PAGE (12%) [41]. The proteins were blotted onto a nitrocellulose membrane and Western blot analyses were performed using a penta-his antibody (1:1000) (Qiagen, Venlo, Netherlands) as described previously [42].

### MtlD activity assay

To determine the activity of the heterologously produced MtlD from *T. kivui*, the NADH dependent fructose-6-phosphate reduction was followed by measuring NADH oxidation at 340 nm (ε = 6,22 mM^−1^ cm^−1^) at 65 °C [35]. Specific activity = ((ΔA_340_ / Δt [min]) × Dilution Factor) / (ε × cm × mg).

### Statistical analysis

The β-glucosidase and MtlD activity analyses were performed in technical and biological triplicates and are presented as the mean ± standard deviation. Statistical analysis was performed using GraphPad Prism 5.0 software (Graphpad Software, Inc., Boston, MA, USA).

### Supplementary Information

**Additional file 1**: **Table S1.**: Plasmids used in this study; **Table S2.**: Primers used in this study; **Table S3.**. Alignment of *mtlD* from *Thermoanaerobacter kivui* with the codon optimized *mtlD* for *Thermus thermophilus;* **Figure S1.**. Verification of the PCR-products and the generated pMKE2-PpilA4-206 and pMKE2-PpilA4-499 plasmids; **Figure S2.**. Verification of the PCR-products and restriction analyses of the generated pLK1-plasmids.

## Data Availability

All data are available.

## Abbreviations

T4P: Type IV pili
BP: Base pairs
RBS: Ribosome binding site
SDS: Sodium dodecyl sulfate
