# Supplementary material for: A temperature dependent pilin promoter for production of thermostable enzymes in Thermus thermophilus
Source: Microb Cell Fact. 2023 Sep 19;22:187. doi: 10.1186/s12934-023-02192-1 (PMC10507856; doi:10.1186/s12934-023-02192-1)
Supplement: Supplementary file 1 — Additional file 1: Table S1.: Plasmids used in this study; Table S2.: Primers used in this study; Table S3.. Alignment of mtlD from Thermoanaerobacter kivui with the codon optimized mtlD for Thermus thermophilus; Figure S1.. Verification of the PCR-products and the generated pMKE2-PpilA4-206 and pMKE2-PpilA4-499 plasmids; Figure S2.. Verification of the PCR-products and restriction analyses of the generated pLK1-plasmids. [file 12934_2023_2192_MOESM1_ESM.docx]

Tab. S1. Plasmids used in this study.

| Plasmid | Description | Source |
| --- | --- | --- |
| pMKE2 | *Thermus* vector encoding a thermostable kanamycin resistance cassette, including a *P_nar_* fragment, a multiple cloning site and a C-terminal hexahistidine tag | (Moreno et al., 2003) |
| pMKE2-*bglT*-his | pMKE2 without the *P_nar_* promoter but containing a promoterless β-glucosidase gene (*bglT*) fused to a C-terminal hexahistidine tag | This study |
| pMKE2-PpilA4_206_for | pMKE2-*bglT*-his including a 206 bp long *P_pilA4_* fragment | This study |
| pMKE2-PpilA4_499_for | pMKE2-*bglT*-his including a 499 bp long *P_pilA4_* fragment | This study |
| *mtlD*_pUC57 | pUC57 including codon optimized *mtlD* without stop codon | Genscript  (Rijswijk, Netherlands) |
| pLK1 | Derived from pMKE2, including a 499 bp long *P_pilA4_* fragment, a multiple cloning site and a hexahistidine tag | This study |
| pLK1-*mtlD*-his | pLK1 containing a codon optimized *mtlD* gene from *Thermoanaerobacter kivui* fused to a C-terminal hexahistidine tag | This study |

Tab. S2. Primers used in this study.

| Primer | Sequence (5′‑3′) | Product length |
| --- | --- | --- |
| bglT_for | ATTATCTAGAAAGCTTGGCAGGAATTCGGAGGCGCATATGACCGAGAACGCCGAAAAATTCC | 1349 bp |
| bglT_rev | AATTATTAGCGGCCGCGGTCTGGGCCCGCGCGATCCG |  |
| pA4_500_for | ATTAAAGCTTACCACGCCCTGGGCTTC | 499 bp |
| pA4_rev | CGGCGAATTCCGCGCCTTCATGTATTG |  |
| pA4_200_for | GCGCAAGCTTAGCTTTCACACCGTTTTCACAC | 206 bp |
| (pA4_rev) | (CGGCGAATTCCGCGCCTTCATGTATTG) |  |
| PpilA4_fwd | CCCCTCTAGAGGATCCCCGGACCACGCCCTGGGCTTCG | 560 bp |
| PpilA4_rev | CAGTCATGCTAGCCATATGGTCATTTCCTCCTAAGATTTCCCGCGC |  |
| pMKE2_fwd | CCATATGGCTAGCATGACTG | 6645 bp |
| pMKE2_rev | CCGGGGATCCTCTAGAGG |  |

Tab. S3. Alignment of *mtlD* from *Thermoanaerobacter kivui* with the codon optimized *mtlD* for *Thermus thermophilus* (* = unchanged base)*.*

| ***mtlD* (optimized)** | ATGCTCAAGGCCGTCCACTTCGGGGCCGGGAACATCGGGCGGGGCTTCATCGGCTACCTGCTCTACAAGAGCGAGTACGAGATCACCTTCGTGGACATCTCCAAGGAGCTCGTGGAGAGC | 120 |
| --- | --- | --- |
| ***mtlD* (wildtype)** | ATGTTAAAAGCGGTTCATTTTGGAGCAGGGAATATAGGCAGAGGATTTATAGGGTATTTGCTTTATAAATCTGAATATGAAATAACTTTTGTTGATATATCTAAAGAATTGGTAGAAAGT | 120 |
| **alignment** | *** * ** ** ** ** ** ** ** ***** ** ** * ** ** ** ** ** **** ** ** ** ** ** ** ** ** ** ** ** ** ** ** * ** ** ** |  |
| ***mtlD* (optimized)** | ATCAACACCTACAAGCGGTACAACGTGATCATCCTCAAGGACAACGTGGAGAAGGAAGAGGTGAAGAACATCAAGGCCATCCACATCGAGGACGAGGAGAACCTCTCCAAGGCCATCGTG | 240 |
| ***mtlD* (wildtype)** | ATAAATACCTATAAAAGATATAATGTGATTATTTTAAAAGATAATGTGGAAAAGGAAGAAGTAAAAAACATCAAAGCTATTCATATCGAAGATGAAGAAAATCTATCCAAGGCTATTGTA | 240 |
| **alignment** | ** ** ***** ** * ** ** ***** ** * ** ** ** ***** ******** ** ** ******** ** ** ** ***** ** ** ** ** ** ******** ** ** |  |
| ***mtlD* (optimized)** | GACGCCGACATCATCACCACCAGCGTGGGGGCCAACAACCTCAAGAGCATCGGCGAGAAGCTCCGGAACTACCTCAAGATCCGCAAGGCCAACATCGACAAGCCCCTCAACATCATGGCC | 360 |
| ***mtlD* (wildtype)** | GATGCAGATATAATTACGACTTCAGTAGGAGCTAATAATCTTAAGAGCATAGGGGAAAAATTAAGAAATTATTTAAAGATAAGAAAAGCTAACATTGATAAACCTTTGAATATAATGGCA | 360 |
| **alignment** | ** ** ** ** ** ** ** ** ** ** ** ** ** ******** ** ** ** * * ** ** * ***** * ** ** ***** ** ** ** * ** ** ***** |  |
| ***mtlD* (optimized)** | TGCGAGAACGCCCTCTTCGCCACCAACATCCTCAAGAACAGCATCCTCGAGAAGGAAGACAAGGACTTCATCGAGTACGTGAACCAGAAGATCGGGTTCCCCAACACCGCCGTGGACCGG | 480 |
| ***mtlD* (wildtype)** | TGCGAAAATGCACTGTTTGCAACAAATATACTAAAAAATAGTATATTGGAAAAAGAGGATAAAGATTTTATTGAGTACGTAAATCAAAAAATTGGTTTTCCTAATACGGCTGTGGACAGG | 480 |
| **alignment** | ***** ** ** ** ** ** ** ** ** ** ** ** ** ** * ** ** ** ** ** ** ** ** ******** ** ** ** ** ** ** ** ** ** ** ****** ** |  |
| ***mtlD* (optimized)** | ATCGTGCCCAACGTGGACATCAAGAAGGAGCTCCCCATCGACGTGGCCGTGGAGGACTTCTACGAGTGGGACATCGACAAGAAGGCCATCATCGGGGACCTCAACATCAAGGGCGTGGAG | 600 |
| ***mtlD* (wildtype)** | ATAGTACCGAATGTAGATATAAAGAAAGAACTGCCAATAGATGTAGCAGTTGAAGATTTTTATGAATGGGATATTGACAAAAAAGCAATTATTGGGGATTTAAATATCAAAGGGGTGGAA | 600 |
| **alignment** | ** ** ** ** ** ** ** ***** ** ** ** ** ** ** ** ** ** ** ** ** ** ***** ** ***** ** ** ** ** ***** * ** ***** ** ***** |  |
| ***mtlD* (optimized)** | CTCGTGGCCGACCTCGAGCCCTACATCGAGCGCAAGCTCTTCCTCCTCAACGGGGCCCACGCCACCACCGCCTACCTCGGGTACCTCAAGGGCTACAAGTACATCCACGAGGCCATCGAG | 720 |
| ***mtlD* (wildtype)** | CTTGTCGCTGACCTTGAACCTTATATAGAGAGAAAATTATTTTTATTAAATGGAGCCCATGCTACCACTGCATATTTAGGATATTTAAAAGGGTATAAATATATACATGAAGCGATTGAG | 720 |
| **alignment** | ** ** ** ***** ** ** ** ** *** * ** * ** * * ** ** ***** ** ***** ** ** * ** ** * ** ** ** ** ** ** ** ** ** ** *** |  |
| ***mtlD* (optimized)** | GACGACTTCATCCGGAACATCGTGTCCGGGATGCAGGAAGAGGCCTCCCTCGCCCTCAGCCGCAAGCACAACATCAAGAAGGACGAGCTCGGCCGGTACGCCTCCAAGGTGATCAAGCGC | 840 |
| ***mtlD* (wildtype)** | GATGATTTTATAAGAAATATAGTGTCTGGAATGCAAGAAGAAGCTTCTCTTGCGCTTTCTAGAAAGCACAATATAAAAAAAGATGAGTTAGGGAGGTATGCAAGTAAGGTAATAAAAAGG | 840 |
| **alignment** | ** ** ** ** * ** ** ***** ** ***** ***** ** ** ** ** ** * ******** ** ** ** ** *** * ** **** ** ***** ** ** * |  |
| ***mtlD* (optimized)** | TTCAAGAACAGCTACCTCAAGGACGAGGTGGTGCGGGTGGGGCGGGAGCCCACCCGGAAGCTCGCCGGCAACGACCGCCTCATGATGCCCGCCAAGTTCTGCTACGAGATCGGGGTGATG | 900 |
| ***mtlD* (wildtype)** | TTTAAAAACTCCTATTTAAAAGACGAAGTTGTAAGAGTAGGGAGAGAGCCTACAAGGAAATTAGCGGGAAATGACCGGTTAATGATGCCTGCTAAGTTTTGCTACGAAATTGGAGTTATG | 900 |
| **alignment** | ** ** *** *** * ** ***** ** ** * ** *** * ***** ** **** * ** ** ** ***** * ******** ** ***** ******** ** ** ** *** |  |
| ***mtlD* (optimized)** | CCCCAGTTCATCCTCTACGGGATCGCCGCCGGCCTCCTCTTCGACTACAAGGAAGACCCCAAGGCCTGCGAGATCCAGAACGACATCAAGAACTTCGGGCTCGAGAAGACCATCTCCAAG | 1080 |
| ***mtlD* (wildtype)** | CCCCAATTTATACTATACGGTATAGCTGCAGGGCTTTTGTTTGATTATAAGGAAGACCCTAAAGCTTGTGAAATCCAAAATGACATTAAAAATTTCGGTTTAGAAAAAACAATAAGCAAA | 1080 |
| **alignment** | ***** ** ** ** ***** ** ** ** ** ** * ** ** ** *********** ** ** ** ** ***** ** ***** ** ** ***** * ** ** ** ** *** |  |
| ***mtlD* (optimized)** | GTGACCGGCCTCGAGGAGAACAGCGGGCTGCTGCAGGAGATCGTCAAGAAGTACAAGGAGCTCAAGGAGGCCTTCGGCAAGCGG--- | 1164 |
| ***mtlD* (wildtype)** | GTTACAGGTTTAGAAGAAAATAGTGGTTTATTGCAGGAAATTGTAAAAAAATATAAAGAATTGAAAGAAGCTTTTGGAAAAAGATGA | 1167 |
| **alignment** | ** ** ** * ** ** ** ** ** * ******* ** ** ** ** ** ** ** * ** ** ** ** ** ** * |  |


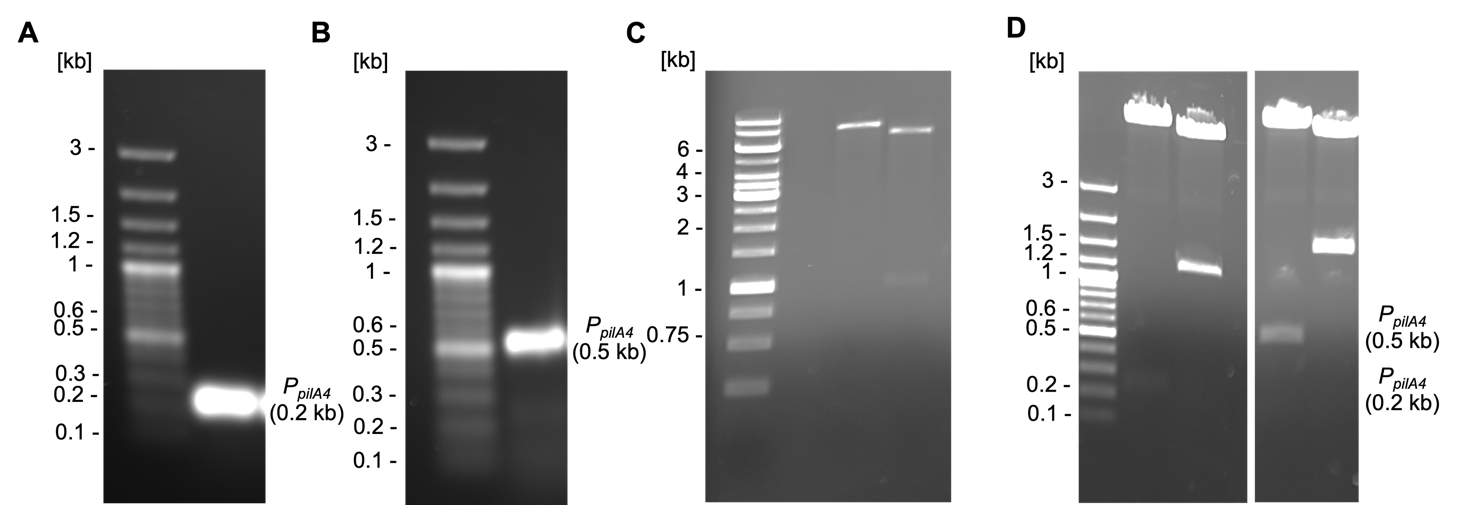


Fig. S1. Verification of the PCR-products and the generated pMKE2-PpilA4-206 and pMKE2-PpilA4-499 plasmids. (A) PCR product of the putative *pilA4* promoter amplified with primers pA4_200_for and pA4_rev. (B) PCR product of the larger DNA-fragment containing putative promoter amplified with primers pA4_500_for and pA4_rev. (C) Restriction of pMKE2-*bglT*-his with *Eco*RI and *Hin*dIII or *Nco*I and *Hin*dIII. (D) Restriction of pMKE2-PpilA4-206 and pMKE2-PpilA4-499 with *Eco*RI and *Hin*dIII or *Nco*I and *Hin*dIII, respectively. The DNA-fragments were separated in 0.8 % agarose gels using TAE buffer (1 h at 100 V). A 100 bp DNA ruler (A, B, D) or 1 kb DNA ruler (C) (Thermo Scientific, Dreieich, Germany) were used as standards.


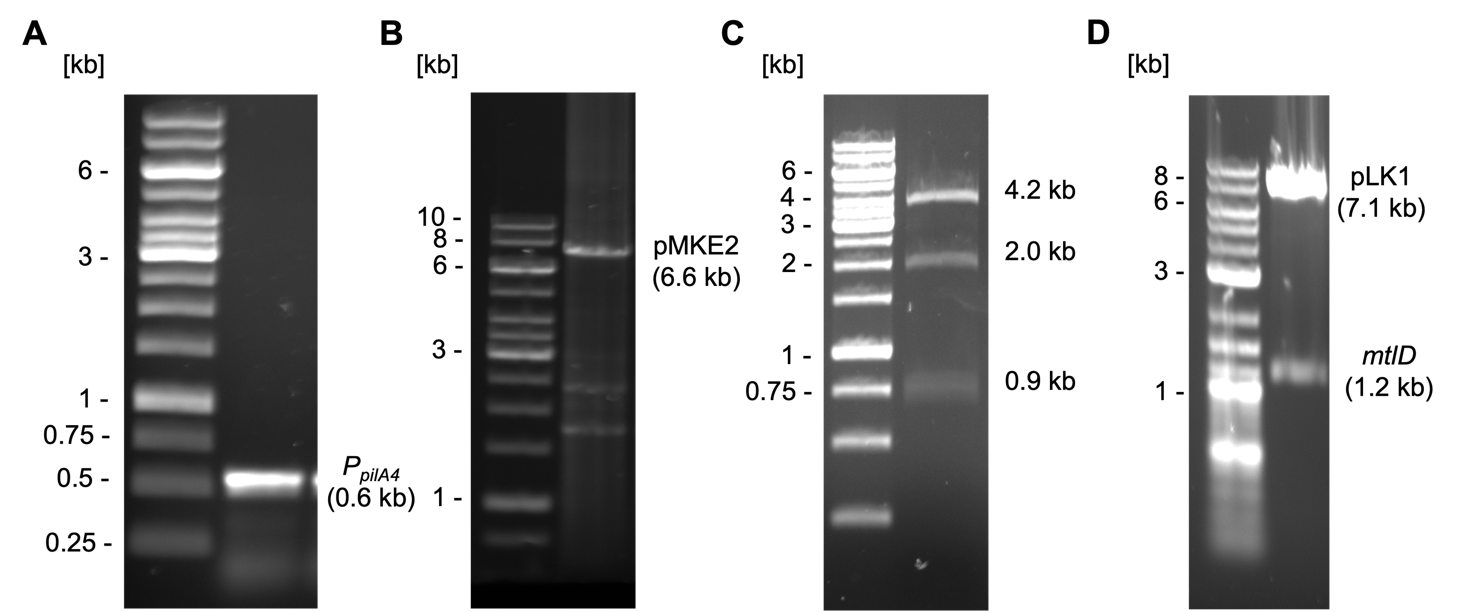


Fig. S2. Verification of the PCR-products and restriction analyses of the generated pLK1-plasmids. (A) PCR product of the *P_pilA4_* fragment amplified from genomic DNA with primers pA4_fwd and pA4_rev. (B) PCR product amplified from the vector backbone with the primers pMKE2_fwd and pMKE2_rev. (C) Restriction of pLK1 with *Bsa*I and *Psi*I. (D) Restriction of pLK1-*mtlD*-his with *Nde*I and *Not*I. The DNA-fragments were separated in 0.8 % agarose gels using TAE buffer (1 h at 100 V). A 1 kb DNA ruler (Thermo Scientific, Dreieich, Germany) was used as standard.
